# Outcome expectancies moderate the association between worry about climate change and personal energy-saving behaviors

**DOI:** 10.1371/journal.pone.0252105

**Published:** 2021-05-26

**Authors:** Thea Gregersen, Rouven Doran, Gisela Böhm, Wouter Poortinga

**Affiliations:** 1 Department of Psychosocial Science, University of Bergen, Bergen, Norway; 2 Centre for Climate and Energy Transformation (CET), University of Bergen, Bergen, Norway; 3 Department of Psychology, Inland Norway University of Applied Sciences, Lillehammer, Norway; 4 Welsh School of Architecture, Cardiff University, Cardiff, Wales, United Kingdom; 5 School of Psychology, Cardiff University, Cardiff, Wales, United Kingdom; Wroclaw University of Economics and Business, POLAND

## Abstract

This paper explores whether efficacy beliefs can alter the relationship between worry about climate change and personal energy-saving behaviors, controlling for climate change beliefs and socio-demographics. For this purpose, we used data from 23 countries that participated in the European Social Survey Round 8 (N = 44 387). Worry about climate change, personal efficacy, personal outcome expectancy, and collective outcome expectancy were each associated with personal energy-saving behaviors concerning either energy curtailment or energy efficiency. The results further show that outcome expectancies moderate the association between worry about climate change and both types of energy behaviors. Worry was more strongly related to energy curtailment behaviors among those with high levels of personal and collective outcome expectancy. A similar pattern was found for energy efficiency behaviors, which were more strongly predicted by worry about climate change when combined with high levels of collective outcome expectancy. These findings are relevant for climate change communication, especially informational campaigns aiming to lower overall household energy use.

## Introduction

In December 2019, the European Commission proposed the European Green Deal as part of a long-term strategy to move towards a circular economy, and eventually reach the goal of net-zero greenhouse gas (GHG) emissions no later than 2050 [1]. Actions outlined to reduce emissions include decarbonizing the energy sector by focusing on low-carbon energy sources and efficient energy use. When it comes to measures that individual households can implement to save energy, the pertinent literature commonly distinguishes between curtailment and efficiency [2, 3]. Energy curtailment refers to actions that save energy by reducing the frequency or intensity of certain behaviors, such as turning off the lights when leaving a room. Energy efficiency-related actions, in contrast, refer to investments in energy-efficient equipment which do not necessarily require behavioral change, such as replacing an old cooling unit in the household. This conceptual distinction has been empirically supported for several household behaviors, including personal attempts to save water [4] and energy [5].

According to statistics provided by the International Energy Agency (IEA), the residential sector accounts for a considerable share of the overall energy consumption in Europe, a pattern that appears relatively stable over time [6]. It follows from this that promoting energy savings at the household level has the potential to significantly reduce GHG emissions and, in turn, help reach the goal of net-zero emissions. Knowledge about individual-level factors that may foster curtailment and/or efficiency behaviors can provide useful insights for targeted communication strategies; and numerous studies have focused on the psychological determinants of such behaviors, both within [e.g., 7–9] and outside [e.g., 10–13] the European context. The investigation reported in this paper is premised on the assumption that people’s motivation to protect themselves against perceived threats, and by extension how they will eventually react to the threatening situation, can be derived from an appraisal of the threat itself and an appraisal of their coping capacity [14, 15]. An increasing volume of empirical studies suggests that the outcomes of these appraisals are not only relevant for explaining coping with individual stressors such as health problems [16, 17], but also for predicting how individuals respond in the face of environmental stressors [18–20].

Threat appraisals—often operationalized as ‘worry’, ‘concern’, or ‘perceived seriousness’—have received considerable research attention with regards to the study of people’s perceptions of climate change [21]. Research focusing specifically on the issue of climate change and self-reported energy-saving behaviors has documented that people who are concerned about climate change report a higher willingness to reduce their energy use [22]. Pertinent literature generally finds a positive relationship between the perceived seriousness of climate change and both curtailment- and efficiency-related actions [23], for example showing that people who rank ‘climate change’ as the most serious issue facing the world are more likely to engage in household energy-saving behaviors, such as buying energy-efficient household appliances [24]. Worry, characterized by the repeated experience of anxiousness or thoughts about a potentially negative event, is considered more personal and experiential than perceived seriousness and concern and thus more likely to motivate mitigative actions [21, 25].

In line with this, recent literature has identified worry about climate change as one individual-level factor that may motivate energy-saving behaviors. Umit, Poortinga, Jokinen and Pohjolainen [26] analyzed data from Round 8 of the European Social Survey (ESS) to explore the role of income on such behaviors while controlling for worry about climate change, among other variables. They reported that those more worried about climate change scored higher on both curtailment-related (i.e., reducing the amount of energy consumed) and efficiency-related (i.e., making financial investments in energy-efficient appliances) actions. Bouman et al. [25] used the same dataset to consider worry as a predictor of energy-saving behaviors and climate policy support. The more individuals expressed worry about climate change, the more likely they were to report engaging in energy-saving behaviors; yet, the direct relationship between the variables was relatively weak [25]. These findings imply that worry about climate change is arguably relevant, but maybe not sufficient, for people to engage in personal energy-saving behaviors. As stated by Steg [27], “people often do not act in line with their concerns, and total household energy use is still rising” (p. 4450). The current paper expands upon the existing literature by exploring whether various forms of efficacy beliefs can explain parts of this gap.

A central aspect of understanding why people engage in or refrain from acting against climate change is their sense of efficacy [28, 29]. The notion of efficacy was originally introduced by Bandura [30], who distinguished between beliefs about one’s ability to perform a specific behavior (personal efficacy) and expectations about whether this behavior will lead to certain outcomes (outcome expectancy). This distinction has been widely used in the psychological literature, for instance in the assumption that perceived efficacy and outcome expectancy may both feed into coping appraisals in the face of health threats [31] and environmental problems [20]. With particular relevance for the present investigation are studies showing that higher levels of personal efficacy are associated with attempts to conserve energy by specifically regulating temperature or generally performing household curtailment behaviors [32] and with a range of other individual pro-environmental [33–35] and adaptation [36] behaviors. Positive relationships between efficacy beliefs and different forms of pro-environmental behaviors have also been reported in empirical investigations with measures combining personal efficacy and outcome expectancy [e.g., 37–40].

Recognizing that any successful mitigative response to climate change necessitates cooperation from across society, there have been calls to investigate efficacy beliefs relating to collective action in addition to, and sometimes instead of, personal efficacy [41, 42]. Studies within this area indicate that believing in a group’s collective ability to achieve desired outcomes relates to public support for policies aimed at reducing carbon emissions [43], public-sphere actions such as voting and protesting [44], household waste management [45], electric vehicle acceptance [46], and intention to reduce plastic use [47]. Some findings indicate that collective efficacy might be more important than personal efficacy in the context of pro-environmental behaviors more generally [18, 19], even though this finding is not always consistent [48, 49].

While there are reasons to assume that being worried will function as a motivation for people to act on climate change [21, 50, 51], believing that one’s actions are insignificant may in the meantime restrain personal engagement, even among those who are aware of the threat climate change poses [52, 53]. Research on fear appeals suggests that perceived risk without a sense of efficacy can lead to denial or disclaiming responsibility rather than taking action [54–56]. Whereas the notion that high levels of perceived risk can hinder action when combined with low levels of efficacy is not always supported [51, 57], it is generally assumed that high levels of efficacy reduce doubts or avoidance in situations where people express a general willingness to act [58–60]. Supporting evidence stems from research showing that informational messages combining high threat with high efficacy are more effective in increasing personal engagement with climate change than messages portraying only the possible negative impacts [61]. One plausible interpretation of this literature is that even though a certain level of concern (or worry) for climate change may facilitate preparedness to take mitigative actions, believing that the proposed strategies are effective and that one has the capacity to implement them, helps enable actual behavioral responses.

### Research aims

Our research draws on cross-national studies supporting a positive relationship between worry about climate change and personal energy-saving behaviors [25, 26]. Building upon these findings, the goal of the current paper is twofold: (i) to establish the relative importance of worry about climate change for explaining self-reported energy curtailment and energy efficiency behaviors, controlling for efficacy beliefs, climate change beliefs, and socio-demographics; (ii) to test whether the relationship between worry about climate change and personal energy-saving behaviors varies as a function of believing that one can perform the behavior (personal efficacy), that it is likely that a large number of people will perform the behavior (collective efficacy), and that this would mitigate climate change either through individual action (personal outcome expectancy) and/or through group action (collective outcome expectancy). We expect that worry will be more strongly related to energy efficiency and curtailment behaviors when combined with the belief that personal and collective behavior change is possible and/or effective for mitigating climate change. Because people living within the same country are likely to share certain characteristics and are thus expected to be somewhat similar in their responses, multilevel models are used to control for possible group effects in energy curtailment and energy efficiency [62].

## Materials and methods

### Data collection

The findings reported in this paper are based on self-report data obtained from *N* = 44 387 respondents from 23 mostly European countries. The data were collected through face-to-face interviews in 2016–2017 as part of the ESS Round 8 [63], which was the first round to include questions on climate change and energy behaviors. Strict random probability sampling was used to draw samples from each country, with participants aged 15 and over. The total sample consists of 48% males and 52% females with a combined mean age of 46.97 years (*SD* = 18.85) when adjusted for post-stratification and population size weights. The study was reviewed and approved by ESS ERIC Research Ethics Committee (REC). In accordance with the ESS ERIC Statutes (Article 23.3), the ESS ERIC subscribes to the Declaration on Professional Ethics of the International Statistical Institute. Written informed consent to participate in the study was given by all participants and was provided by the participant’s legal guardian/next of kin if the respondent was under 16 years of age at the time of the interview. More detailed information about the data is available in the documentation report [64].

### Measurements

Two types of energy behaviors were included as dependent variables in the analyses, energy curtailment behaviors, and energy efficiency behaviors [2, 3]. Energy curtailment behaviors were measured by asking “There are some things that can be done to reduce energy use, such as switching off appliances that are not being used, walking for short journeys, or only using the heating or air conditioning when really needed. In your daily life, how often do you do things to reduce your energy use?”, with response categories 1 (*Never*), 2 (*Hardly ever*), 3 (*Sometimes*), 4 (*Often*), 5 (*Very often*), and 6 (*Always*). Energy efficiency behaviors were assessed with the question “If you were to buy a large electrical appliance for your home, how likely is it that you would buy one of the most energy efficient ones?”, measured on a scale from 0 (*Not likely at all*) to 10 (*Extremely likely*). There were *n* = 551 (for curtailment) and *n* = 1 111 (for efficiency) missing values.

The independent variables consisted of worry about climate change and four efficacy beliefs. Worry about climate change was measured by asking respondents to answer the question “How worried are you about climate change?” from 1 (*Not at all worried*), 2 (*Not very worried*), 3 (*Somewhat worried*), 4 (*Very worried*) to 5 (*Extremely worried*). The item had *n* = 1 733 missing values.

The four efficacy beliefs referred to (a) personal efficacy, (b) personal outcome expectancy, (c) collective efficacy, and (d) collective outcome expectancy. Personal efficacy was measured with the question “Overall, how confident are you that you could use less energy than you do now?” with a response scale ranging from 0 (*Not at all confident*) to 10 (*Completely confident*) and *n* = 952 missing observations. The question “How likely do you think it is that limiting your own energy use would help reduce climate change?” was used as an indicator of personal outcome expectancy. Collective efficacy was assessed with the question “How likely do you think it is that large numbers of people will actually limit their energy use to try to reduce climate change?”. Finally, the following question captured the level of collective outcome expectancy: “Now imagine that large numbers of people limited their energy use. How likely do you think it is that this would reduce climate change?”. The last three questions were answered on a scale from 0 (*Not at all likely*) to 10 (*Extremely likely*), with *n* = 2 733 (for personal outcome expectancy), *n* = 2 977 (for collective efficacy), and *n* = 3 255 (for collective outcome expectancy) missing values.

Covariates in the analyses included climate change beliefs and a number of socio-demographic variables. Climate change beliefs, previously shown to be related to worry about climate change in the ESS [65], were captured with two questions. The question “Do you think that climate change is caused by natural processes, human activity, or both?”, with answer categories 1 (*Entirely by natural processes*), 2 (*Mainly by natural processes*), 3 (*About equally by natural processes and human activity*), 4 (*Mainly by human activity*), or 5 (*Entirely by human activity*), was asked to assess respondents’ beliefs about the anthropogenic causation of climate change. There was a total of *n* = 2 502 missing values to this question, including the response option labeled “I don´t think the climate is changing”. Evaluation of the seriousness of climate change consequences was assessed with the question “How good or bad do you think the impact of climate change will be on people across the world?”. The question was originally answered on an 11-point scale ranging from 0 (*Extremely bad*) to 10 (*Extremely good*), which was later reversed and dichotomized to 0 (*Belief that the impacts will be good or neutral*), including answers from 5 to 10, and 1 (*Belief in mostly bad impacts*), including answers from 0 to 4. The variable was dichotomized in order to distinguish those who believe in mostly negative impacts of climate change from those who do not, following similar procedures as in Gregersen et al. [65].

Household income was categorized from the 1^st^ to 10^th^ decile. It should be noted that this variable had quite a lot of missing observations (*n* = 7 942). Of these, *n* = 4 990 missing observations were due to refusal to answer the question, while the rest compromised “don’t know” (*n* = 2902) and missing data without an assigned explanation (*n* = 50). Political orientation was assessed by asking respondents “In politics people sometimes talk of ‘left’ and ‘right’. Using this card, where would you place yourself on this scale, where 0 means the left and 10 means the right?”. The variable had 5 804 missing observations. Age was categorized into ten-year intervals and gender was dichotomized into male (0) and female (1). Education was measured based on the ESS version of the ISCED (International Standard Classification of Education) categorization: 1 (*ES-ISCED I /less than lower secondary*), 2 (*ES-ISCED II/lower secondary*), 3 (*ES-ISCED IIIb/lower-tier upper secondary*), 4 (*ES-ISCED IIIa/upper-tier upper secondary*), 5 (*ES-ISCED IV/advanced vocational/sub-degree*), 6 (*ES-ISCED V1/lower tertiary education/BA level*), 7 (*ES-ISCED V2/higher tertiary education/> = MA level*).

Further descriptive information can be found in Table 1. Correlations between the main variables are reported in Table 2.

**Table 1:** Descriptive statistics for the variables in the study.

Individual level (*N* = 44 387)	*M*	*SD*	Min	Max
Energy curtailment behavior (1 = Never; 6 = Always)	4.09	1.28	1	6
Energy efficiency behavior (0 = Not at all likely; 10 = Extremely likely)	7.53	2.43	0	10
Worry about climate change (1 = Not at all worried; 5 = Extremely worried)	3.06	0.94	1	5
Personal efficacy (0 = Not at all confident; 10 = Completely confident)	5.87	2.62	0	10
Personal outcome expectancy (0 = Not at all likely; 10 = Extremely likely)	4.35	2.58	0	10
Collective efficacy (0 = Not at all likely; 10 = Extremely likely)	4.05	2.15	0	10
Collective outcome expectancy (0 = Not at all likely; 10 = Extremely likely)	5.51	2.34	0	10
Household income (1 = 1st decile; 10 = 10th decile)	5.36	2.76	1	10
Climate change attribution (1 = Entirely natural processes; 5 = Entirely human activity)	3.42	0.83	1	5
Climate change impact (0 = Extremely good; 10 = Extremely bad)	6.80	2.19	0	10
Age	46.97	18.85	15	100
Gender (Female)	0.52	0.50	0	1
Education	3.78	1.82	1	7

*Note*. All variables are weighted with a combination of post-stratification weights and population weights. The variables are presented in their original scales, except that climate change impact has been reversed.

**Table 2:** Correlation matrix for the main predictors in the study (weighted).

	Energy curtailment behavior	Energy efficiency behavior	Worry about climate change	Personal efficacy	Personal outcome expectancy	Collective efficacy	Collective outcome expectancy	Climate change attribution	Climate change impact	Political orientation	Age	Gender (Female)	Household Income	Education
Energy curtailment behavior	1.00													
Energy efficiency behavior	0.36**	1.00												
Worry about climate change	0.23**	0.21**	1.00											
Personal efficacy	0.21**	0.22**	0.17**	1.00										
Personal outcome expectancy	0.14**	0.14**	0.24**	0.27**	1.00									
Collective efficacy	0.07**	0.05**	0.11**	0.17**	0.44**	1.00								
Collective outcome expectancy	0.15**	0.15**	0.28**	0.27**	0.45**	0.35**	1.00							
Climate change attribution	0.06**	0.08**	0.30**	0.09**	0.11**	0.03**	0.21**	1.00						
Climate change impact	0.09**	0.11**	0.29**	0.04**	-0.04**	-0.16**	0.07**	0.24**	1.00					
Political orientation	-0.06**	-0.02**	-0.11**	-0.01	-0.01	0.04**	-0.04**	-0.10**	-0.12**	1.00				
Age	0.18**	0.12**	-0.01*	-0.06**	-0.02**	0.05**	-0.04**	-0.07**	-0.04**	0.04**	1.00			
Gender (Female)	0.04**	0.00	0.05**	-0.04**	0.01**	0.03**	0.04**	-0.01**	-0.02**	-0.02**	0.06**	1.00		
Household income	-0.04**	0.09**	0.03**	0.10**	0.01*	-0.05**	0.02**	0.06**	0.04**	0.01**	-0.20**	-0.10**	1.00	
Education	0.01	0.06**	0.06**	0.06**	0.00	-0.04**	0.02**	0.04**	0.07**	-0.04**	-0.17**	0.01	0.38**	1.00

*Note*. Pairwise correlation coefficients. Weighted with a combination of post-stratification weights and population weights. All variables are used in their original scales, except that climate change impact has been reversed.

*p < .05 (two-tailed)

**p < .01 (two-tailed)

***p < .001 (two-tailed).

### Statistical analysis

Due to the nested structure of the ESS Round 8 data, the associations between worry about climate change and personal energy-saving behaviors were analyzed by fitting linear two-level (individual and country) multilevel models in Stata 16 using the mixed command. As we were interested in the overall effect of the variables and in generalizing the results to a broader population, the role of the country-level residuals was to help estimate standard errors correctly. We conducted multiple regressions to allow us to measure the effect of each predictor while controlling for the other relevant variables.

We started by fitting an unconditional null model, followed by a random-intercept model including all predictors (Model 1), and lastly adding the four interaction terms (Model 2). This procedure was conducted separately for energy curtailment and energy efficiency behaviors, as these were predicted in separate models. All models were estimated with maximum likelihood and compared with likelihood ratio tests. The margins and marginsplot commands were used to interpret the interaction effects. Except for gender and beliefs about climate change consequences, which were both dichotomized, all other variables were treated as continuous and grand-mean centered in the main models. Standardized versions of the variables were used in complementary models to allow us to compare the distinct influence each predictor had on the outcomes. Standardization can influence the interpretation of variance [62], and was therefore avoided in the models presented in the multilevel regression tables. Proportional reduction in variance (PRV), calculated by comparing the explained variance of the main effects models with and without the worry item, was used as a second indication of the effect size of worry about climate change. Results from the standardized models and PRV calculations are presented below. Finally, *Pseudo R*^*2*^ was used to indicate the variance explained by all variables combined, following recommendations by Rabe-Hesketh and Skrondal [66]. *Pseudo R*^*2*^ was calculated by comparing the total residual variance of the null model to the residual variance of the fitted models.

## Results

**Fig 1:**
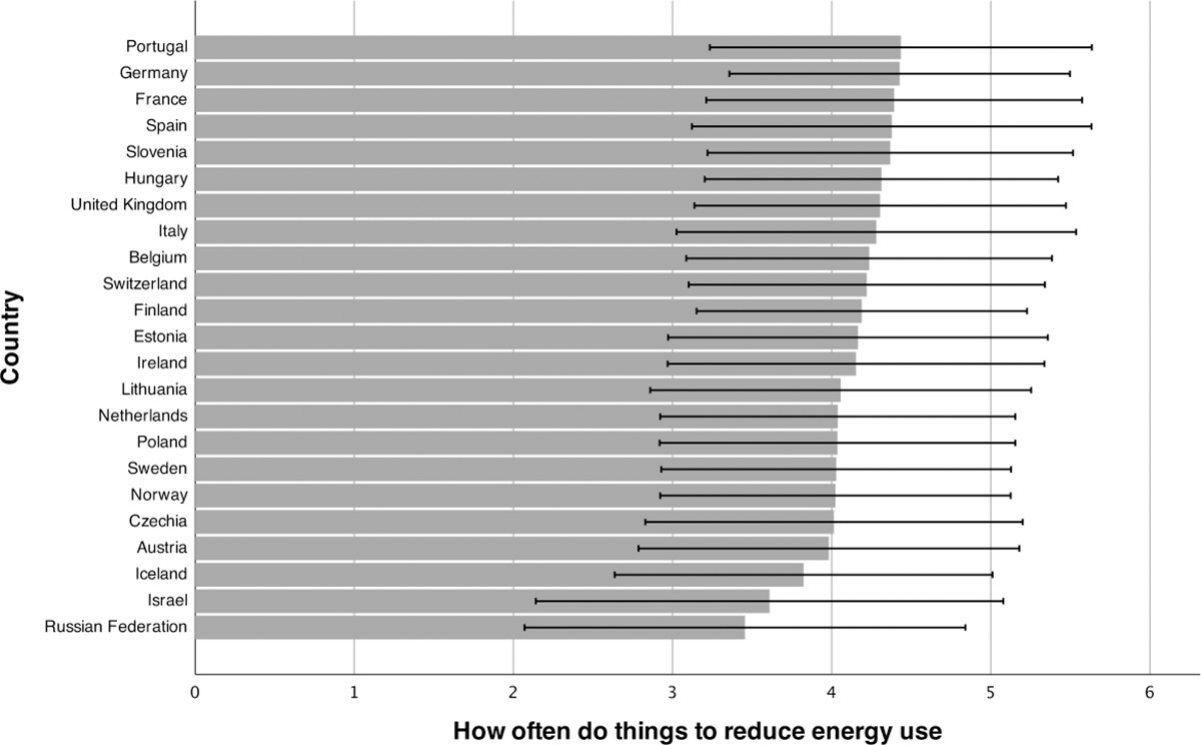
Mean energy curtailment behavior. Note. Means weighted with a combination of post-stratification weights and population weights. The figure includes +/- 1 SD.

**Fig 2:**
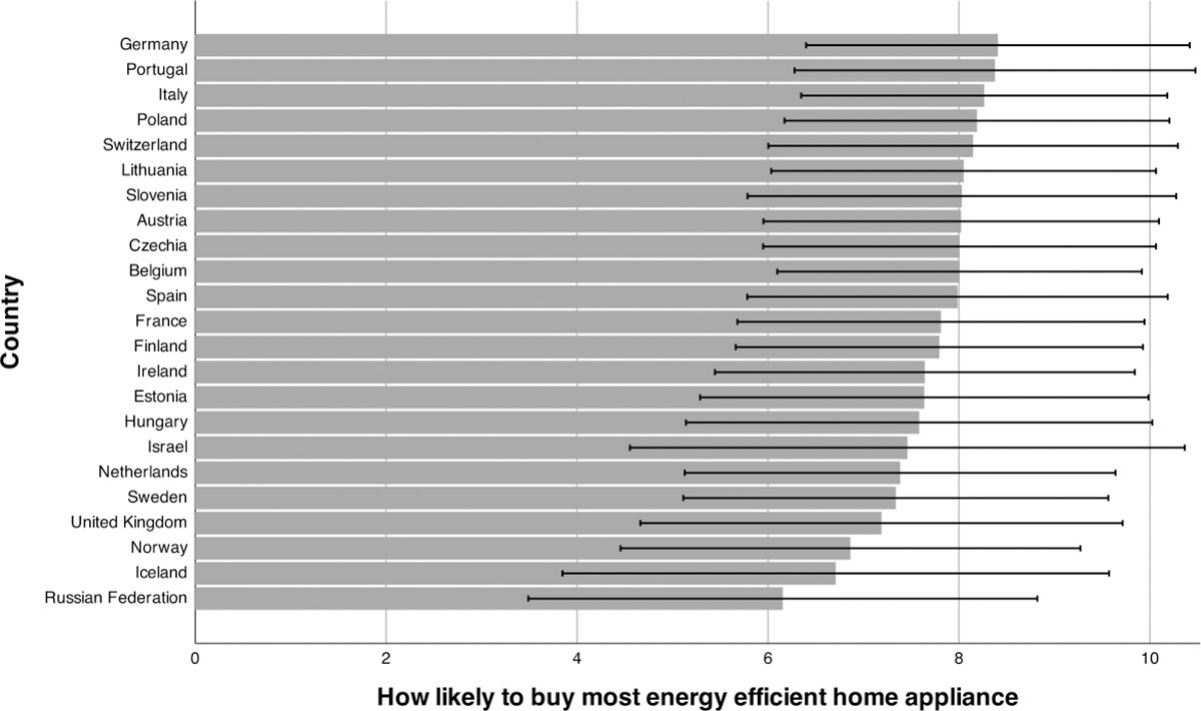
Mean energy efficiency behavior. Note. Means weighted with a combination of post-stratification weights and population weights. The figure includes +/- 1 SD.

Fig 1 shows the country-specific means for energy curtailment behaviors, and Fig 2 the country-specific means for energy efficiency behaviors, including standard deviations. In total, 68% of respondents answered that they often, very often, or always do things to reduce their energy use. Furthermore, about 80% answered above the midpoint of the 11-point scale when asked how likely they are to buy energy-efficient appliances, with 27% answering at the endpoint (“extremely likely”). The weighted correlation between the two outcomes is *r* = .36, which is usually considered a moderate effect [67].

Intraclass correlation (ICC), calculated as σ^2^_country_/(σ^2^_country_ + σ^2^_individual_), was used to explore the homogeneity within countries regarding energy behaviors [62, 68]. In the unconditional model, the country level explained about 3%, ICC = 0.03, 95% CI [0.02,0.06], of the variation in energy curtailment behaviors and 6%, ICC = 0.06, 95% CI [0.03, 0.10], of the variation in energy efficiency behaviors. Even though the variance explained at the country level was sparse, we decided on multi-level models rather than single-level models to reduce the likelihood of Type I error. This follows the idea that clustering should be accounted for independently of ICC levels [69].

Tables 3 and 4 present the unstandardized coefficients of the main effects (Model 1) for curtailment and efficiency behaviors, respectively. Results from standardized versions of the model show that worry was one of the strongest predictors, *β* = .19 (*p* < .001), of energy curtailment behaviors, together with age, *β* = .18 (*p* < .001). Also, personal efficacy, *β* = .05 (*p* < .001); personal outcome expectancy, *β* = .04 (*p* < .001); and collective outcome expectancy, *β* = .05 (*p* < .001); were each positively associated with self-reported curtailment behaviors. Energy curtailment was further associated with higher levels of education, *β* = .08 (*p* < .001); lower household income, *β* = -.06 (*p* < .001); a self-identified left-leaning political orientation, *β* = -.04 (*p* < .001); and with less belief in anthropogenic causes of climate change, *β* = -.02 (*p* = .006). Women reported a higher frequency of curtailment than men, *β* = .04 (*p* < .001). Including all covariates led to an improvement in model fit compared to the unconditional model, *χ*^*2*^(12) = 2009.30, *p* < .001, and about 7% of the residual variance in energy curtailment behaviors was explained (*Pseudo R*^*2*^ = 0.07). Worry about climate change accounts for about 3% (PRV = 0.03) of this variance.

**Table 3:** Model results–Energy curtailment behavior.

	Null Model	Model 1 (Main effects)	Model 2 (Interactions)
	*B (SE)*	*B (SE)*	*B (SE)*
Fixed coefficients			
Intercept	4.19 (0.04)	4.11 (0.04)	4.01 (0.04)
Worry about climate change		0.21 (0.01)***	0.21 (0.01)***
Personal efficacy (PE)		0.02 (0.00)***	0.02 (0.00)***
Personal outcome expectancy (POE)		0.01 (0.00)***	0.01 (0.00)***
Collective efficacy (CE)		-0.00 (0.00)	-0.00 (0.00)
Collective outcome expectancy (COE)		0.02 (0.00)***	0.02 (0.00)***
Worry about climate change × PE			-0.00 (0.00)
Worry about climate change × POE			0.01 (0.00)*
Worry about climate change × CE			0.00 (0.00)
Worry about climate change × COE			0.01 (0.00)**
Climate change attribution		-0.03 (0.01)**	-0.02 (0.01)*
Climate change impact (Bad)		0.02 (0.02)	0.02 (0.02)
Political orientation		-0.02 (0.00)***	-0.02 (0.00)***
Age		0.01 (0.00)***	0.01 (0.00)***
Gender (Female)		0.07 (0.01)***	0.07 (0.01)***
Household income		-0.02 (0.00)***	-0.02 (0.00)***
Education		0.05 (0.00)***	0.04 (0.00)***
Random parameters			
Level 2: Country(var)	0.05 (0.01)	0.03 (0.01)	0.03 (0.01)
Level 1: Individual(var)	1.30 (0.01)	1.22 (0.01)	1.21 (0.01)
Log likelihood	-45770.793	-44766.145	-44748.805
AIC	91547.59	89562.29	89535.61
Variance explained	ICC = 0.03, 95% CI [0.02,0.06]	*Pseudo R*^*2*^ = 0.07	*Pseudo R*^*2*^ = 0.07
		*R*_*2*_^*2*^ = 0.03	*R*_*2*_^*2*^ = 0.03
		*R*_*1*_^*2*^ = 0.06	*R*_*1*_^*2*^ = 0.06

*Note*. Total R-squared and separate reduction in variance are calculated following the method used in Rabe-Hesketh and Skrondal [66]. *N* = 29 492 individuals, *N* = 23 countries. All variables are grand-mean centered, except gender (0 = Male; 1 = Female) and climate change impact (0 = Good; 1 = Bad). Unweighted.

*p < .05

**p < .01

***p < .001.

**Table 4:** Model results–Energy efficiency behavior.

	Null Model	Model 1 (Main effects)	Model 2 (Interactions)
	*B (SE)*	*B (SE)*	*B (SE)*
Fixed coefficients			
Intercept	7.83 (0.11)	7.64 (0.11)	7.64 (0.11)
Worry about climate change		0.30 (0.02)***	0.30 (0.02)***
Personal efficacy (PE)		0.08 (0.01)***	0.08 (0.01)***
Personal outcome expectancy (POE)		0.03 (0.01)***	0.03 (0.01)***
Collective efficacy (CE)		-0.01 (0.01)	-0.01 (0.01)
Collective outcome expectancy (COE)		0.05 (0.01)***	0.05 (0.01)***
Worry about climate change × PE			-0.01 (0.00)
Worry about climate change × POE			-0.00 (0.01)
Worry about climate change × CE			-0.01 (0.01)
Worry about climate change × COE			0.01 (0.01)*
Climate change attribution		-0.00 (0.02)	-0.00 (0.02)
Climate change impact (Bad)		0.06 (0.03)*	0.06 (0.03)*
Political orientation		-0.00 (0.01)	-0.00 (0.01)
Age		0.02 (0.00)***	0.02 (0.00)***
Gender (Female)		0.16 (0.02)***	0.16 (0.02)***
Household income		0.04 (0.00)***	0.04 (0.00)***
Education		0.08 (0.01)***	0.08 (0.01)***
Random parameters			
Level 2: Country	0.27 (0.08)	0.26 (0.08)	0.26 (0.08)
Level 1: Individual	4.52 (0.04)	4.20 (0.03)	4.20 (0.03)
Log likelihood	-64053.153	-62969.588	-62965.489
AIC	128112.3	125969.2	125969
Variance explained	ICC = 0.06, 95% CI [0.03,0.10]	*Pseudo R*^*2*^ = 0.07	*Pseudo R*^*2*^ = 0.07
		*R*_*2*_^*2*^ = 0.04	*R*_*2*_^*2*^ = 0.04
		*R*_*1*_^*2*^ = 0.07	*R*_*1*_^*2*^ = 0.07

*Note*. Total R-squared and separate reduction in variance are calculated following the method used in Rabe-Hesketh and Skrondal [66]. *N* = 29 448 individuals, *N* = 23 countries. All variables are grand-mean centered, except gender (0 = Male; 1 = Female) and climate change impact (0 = Good; 1 = Bad). Unweighted.

*p < .05

**p < .01

***p < .001.

Regarding purchases of energy-efficient appliances, the strongest association was with age, *β* = .37 (*p* < .001); followed by worry, *β* = .28 (*p* < .001). Personal efficacy, *β* = .20 (*p* < .001); personal outcome expectancy, *β* = .08 (*p* < .001); and collective outcome expectancy, *β* = .13 (*p* < .001), each predicted a higher likelihood of purchasing energy-efficient appliances. Respondents with higher levels of education also reported a higher likelihood of buying efficient appliances, *β* = .15 (*p* < .001), as did women, *β* = .08 (*p* < .001), those with higher household income, *β* = .11 (*p* < .001), and those believing in negative impacts of climate change, *β* = .03 (*p* = .039). According to the PRV calculation, worry about climate change explained less than 2% of the variance in energy efficiency behaviors (PRV = 0.02). The model explained approximately 7% of the residual variance (*Pseudo R*^*2*^ = 0.07) and was an improvement compared to the unconditional model, *χ*^*2*^(12) = 2167.13, *p* < .001.

**Fig 3:**
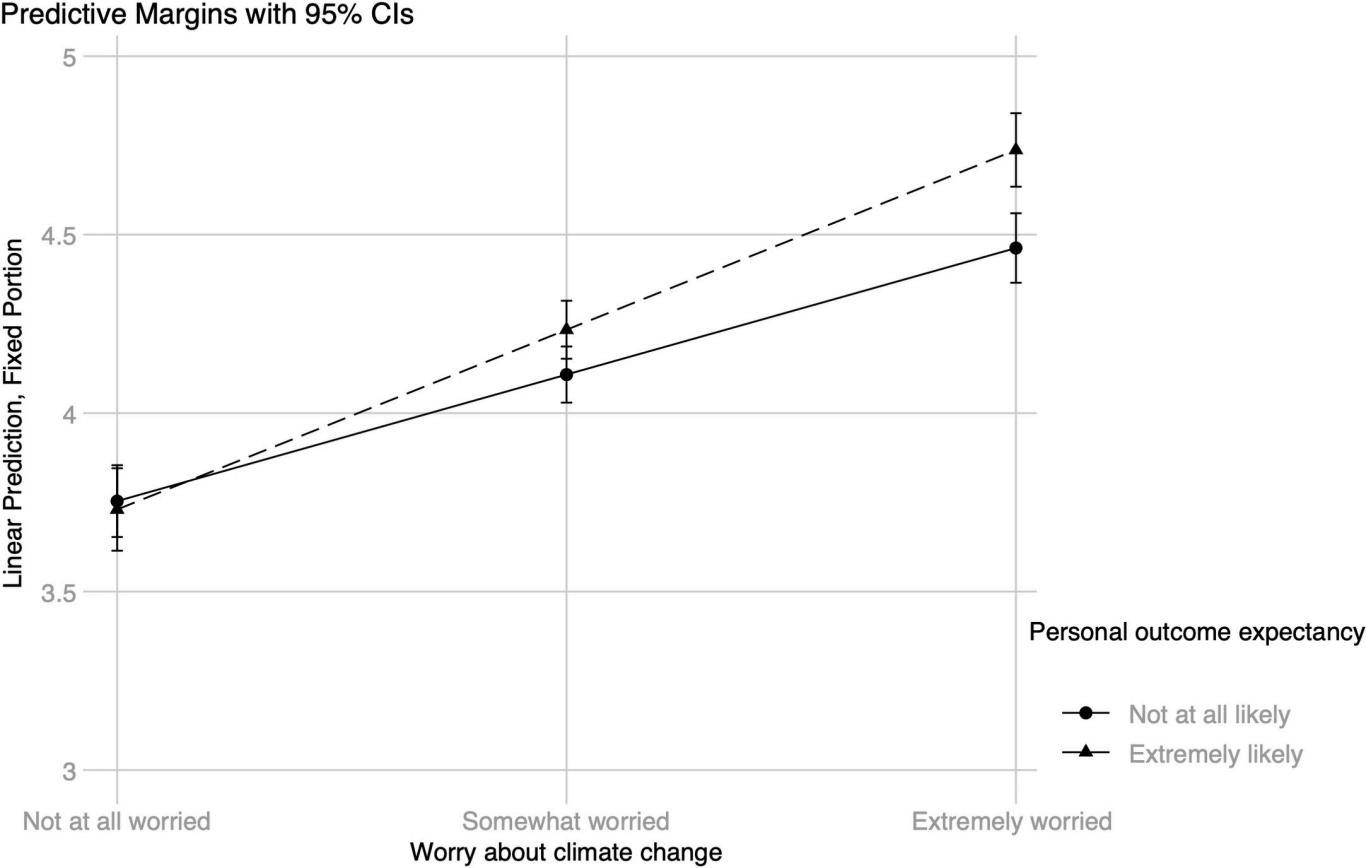
Energy curtailment behavior: Worry about climate change × personal outcome expectancy.

**Fig 4:**
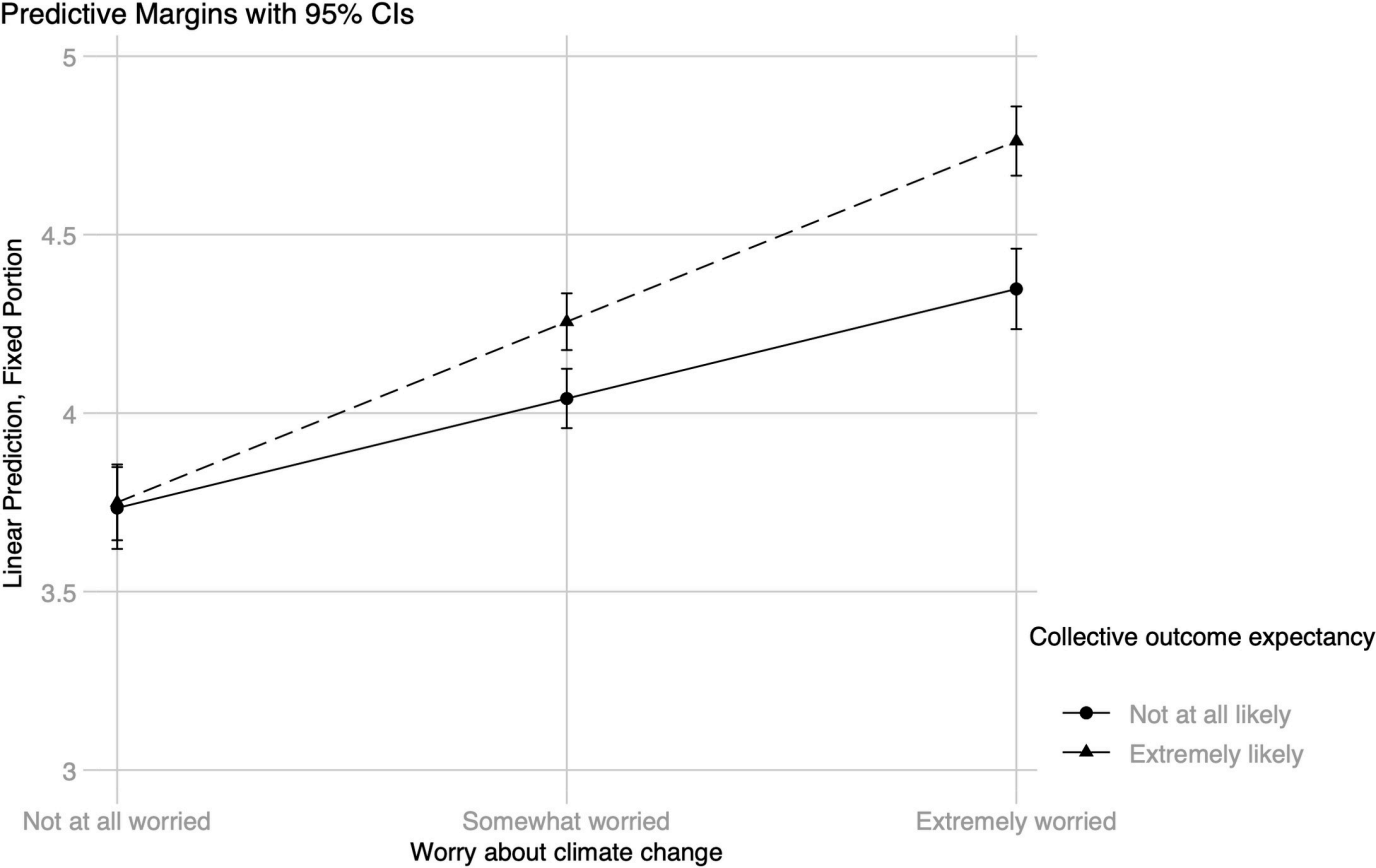
Energy curtailment behavior: Worry about climate change × collective outcome expectancy.

Four interactions between worry and efficacy beliefs were added in Model 2, which is presented in Table 3 for curtailment and in Table 4 for efficiency behaviors. For the model including energy curtailment behavior as the dependent variable, there were two statistically significant interactions (see Table 3). The simple slopes for worry were statistically significant at both low, *B* = .18 (.02), *z* = 11.53, *p* < .001, 95% CI [0.15, 0.21], and high, *B* = .25 (.02), *z* = 13.48, *p* < .001, 95% CI [0.22, 0.29], levels of personal outcome expectancy; the same pattern was found at low, *B* = .15 (.02), *z* = 7.74, *p* < .001, 95% CI [0.11, 0.19], and high, *B* = .25 (.02), *z* = 15.69, *p* < .001, 95% CI [0.22, 0.28], levels of collective outcome expectancy. However, an inspection of the interaction plots shows that worry about climate change had a stronger association with curtailment for those with high levels of personal outcome expectancy (see Fig 3) as well as those with high levels of collective outcome expectancy (see Fig 4). The difference between people with high versus low levels of personal outcome expectancy seems to occur only at high levels of worry, while the difference between high versus low levels of collective outcome expectancy is present at both moderate and high levels of worry. The reported level of outcome expectancy, whether personal or collective, does not seem to make a difference among individuals who are not at all worried about climate change. Including the interactions significantly improved the model with curtailment behavior as the criterion, *χ*^*2*^(4) = 34.68, *p* < .001.

**Fig 5:**
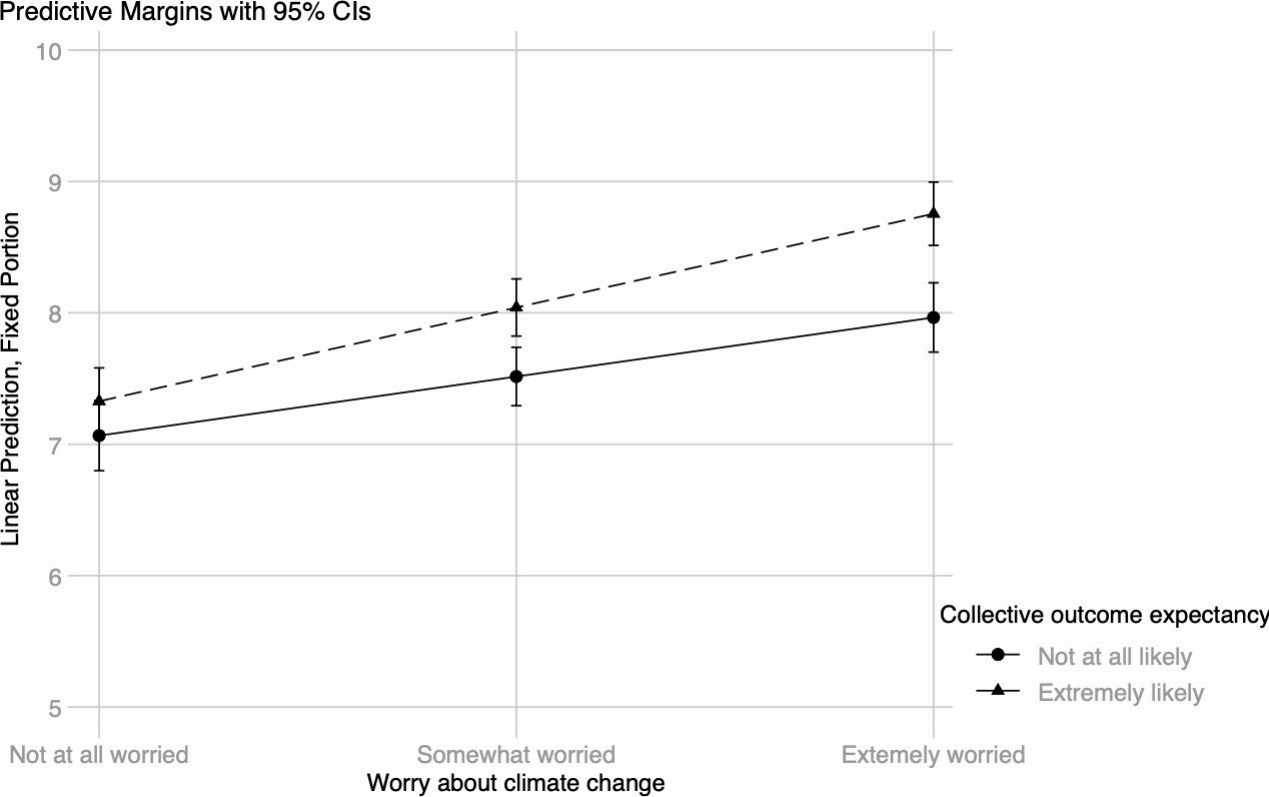
Energy efficiency behavior: Worry about climate change × collective outcome expectancy.

For the model with energy efficiency behaviors as the dependent variable, only one interaction was statistically significant (see Table 4). The simple slopes show a positive relationship between worry and efficiency behavior at both low, *B* = .22 (.04), *z* = 6.08, *p* < .001, 95% CI [0.15, 0.30], and high, *B* = .36 (.03), *z* = 11.86, *p* < .001, 95% CI [0.30, 0.42], levels of collective outcome efficacy. Still, the plot displayed in Fig 5 indicates that the relationship is stronger for those who score high on collective outcome expectancy at both moderate and high levels of worry about climate change. Again, there is no difference between those with high versus low levels of collective outcome expectancy among individuals who are not at all worried about climate change. Including the interactions did not statistically significantly improve the model fit, *χ*^*2*^(4) = 8.20, *p* = .085.

All main coefficients stay highly similar from Model 1 to Model 2; and the variables continue to explain about 7% of the variance in each of the two types of personal energy-saving behaviors (*Pseudo R*^*2*^ = 0.07). Additionally, the estimated intraclass correlations do not change substantially from the unconditional model to the final model and indicate that unobserved characteristics at the country level can explain about 3% of the variance in energy curtailment behaviors and about 6% of the variance in energy efficiency behaviors.

## Discussion

Our results show that worry about climate change was an important predictor of individuals engaging in both energy curtailment and energy efficiency behaviors, compared to most of the other variables included in the study. They further show that both curtailment and efficiency behaviors were more likely to be reported when such behaviors were perceived to be possible (high personal efficacy) and effective in mitigating climate change when employed alone (personal outcome expectancy) or as a collective (collective outcome expectancy). This supports previous findings establishing that different efficacy constructs can be empirically distinguished in terms of their contribution to pro-environmental behaviors [e.g., 43, 44, 70]. The distinct effect size of each of the significant efficacy constructs was highly similar when predicting energy curtailment behaviors. In contrast, the effect sizes of the efficacy constructs differed with respect to purchasing energy-efficient appliances. Personal efficacy showed the strongest association, followed by collective outcome expectancy and finally personal outcome expectancy. Notably, personal efficacy had the weakest association with the other efficacy items (see Table 2). Collective efficacy, operationalized as believing that many people will limit their energy use to reduce climate change, failed to show any statistically significant associations in predicting energy saving behaviors. Other measurements of collective efficacy have also shown non-significant [44] or weak [43] effects in previous studies.

The present research was based on the idea that efficacy beliefs may interact with worry about climate change in shaping behavioral responses to environmental problems. Contrary to our expectations, only outcome expectancies showed any significant moderating effects in our models. Examples of reasons for low scores on the outcome expectancy measurements would be believing one’s individual energy-saving efforts to be insignificant compared to the non-action of other individuals (low personal outcome expectancy) or that collective energy-saving is trivial in the face of emissions from big companies (low collective outcome expectancy). Our results support the assumption that worrying about climate change is more strongly related to energy curtailment behavior for those with high levels of personal and collective outcome expectancy, and that energy efficiency behavior is best predicted by a combination of high worry and high levels of collective outcome expectancy. A possible reason for finding collective outcome expectancy to be the most consistent moderator might be the global scale and inherently collective nature of climate change [41, 42]. Our research focuses on the global effects of energy-saving as seen by the public in European countries, which allows for the possibility that moderation effects in regards to the remaining efficacy measures can be found in other contexts. Previous research supports this view insofar that household energy behaviors can have somewhat different predictors across countries [25] and cities [10].

High worry appears to be positively associated with energy behaviors at both low and high levels of outcome expectancy. Though it should be noted that the general measurement of worry about climate change used in the current study differs from the immediate fear appeals used in many experimental studies [e.g., 71], the finding support that high levels of perceived risk do not seem to have a negative effect (‘backfire’) [51, 57]. High levels of outcome expectancy seem to have the potential to reinforce the effect of high worry, but not make a difference if people are not at all worried about climate change. The lack of effect of outcome expectancies at low levels of worry indicates that the motivational aspect of worrying about climate change is additionally necessary to create intent and desire to engage in personal energy-saving behaviors. Without the arousal introduced by feeling worried and the recognition of climate change as a risk, the notion that individual or collective energy-saving behaviors would help reduce climate change might not be seen as personally relevant. This is in line with seeing concern as a necessary pre-condition [60]. Based on our findings, one strategy for climate change communication might be to continue to inform people about the risks associated with continued global warming [72], while also focusing on the mitigative potential of individual and collective actions [73].

### Limitations

When investigating complex human behavior, small effect sizes are expected. While acknowledging that small effects can still be highly relevant and important to the field [74], it should be noted that our models including all individual-level variables and interactions account for only about 7% of the variance in both energy-saving behaviors. According to this and our other effect size measures, the relationship between worry about climate change and energy-saving behaviors is rather weak. This is in line with prior literature pointing to a gap between an expressed concern about climate change and the individual willingness to act [75, 76].

There may be several reasons why we find relatively weak effects in the current study. First, our measurement of worry may not have captured the state we were interested in with perfect validity. One item asking the respondent ‘how worried’ he or she is about climate change might prime a short-term, passive agreement indicating awareness of the issue rather than the active, personal emotion we sought to capture. In future research, a better qualitative, methodological understanding of people’s responses to this question is necessary. For example, researchers could use cognitive interviewing to determine how people read and perceive this and similar items and what they associate with the term ‘being worried’ in a survey context. Second, the measurement of worry focuses on climate change in general rather than on the issue of energy consumption in particular. It could be that energy behaviors would be more strongly related to behavior-specific concerns rather than the more generalized climate change worry [for supporting evidence, see 11, 77]. Third, threat and coping appraisals do not appear to be sufficient for motivating individuals to engage in energy saving at the household level. Instead, the comparatively weak effects point to the importance of considering other factors not included in this study, such as norms, habits, and structural constraints [77, 78]. Finally, even when climate change is perceived to be a threat that requires action, energy behaviors may not be seen as particularly relevant or effective in this regard. If this is the case, one might expect the interaction effects between worry and outcome expectancy to be stronger. However, the questions used to measure personal and collective outcome expectancy ask only whether limiting energy use would help reduce climate change, not how big the impact would be. People might still perceive energy savings to have quite a limited effect compared to other actions. Increased information about the comparative effectiveness of energy curtailment and energy efficiency behaviors or about how they contribute to a sustainable lifestyle might be helpful if the goal is to change people’s perceptions of such behaviors.

The study has some additional limitations regarding the measurements used to capture energy behaviors. First, whether people who express willingness to engage in personal energy-saving behaviors implement these behaviors in their everyday lives cannot be answered based on the present data. Previous research employing meta-analytic techniques has found only a moderate correlation between self-reported and objective measures of pro-environmental behavior, leaving 79% of the variance unexplained [79]. Second, the analyses relied upon single-item measures of energy curtailment and energy efficiency behaviors, both of which were formulated in rather broad terms. For example, the item assessing curtailment included an array of example behaviors spanning from switching off unused appliances and walking for shorter journeys to adjusting the heating or air conditioning. This might be problematic since research has shown that the acceptability of energy-saving behaviors is generally higher for home-related behaviors compared to transport-related behaviors [80] and that certain subgroups (poorer households) are more likely to use less money on transport and space heating without being more likely to turn off lights, turn down heat or switch appliances to standby on a daily basis [81]. Consequently, the measurements utilized might be inaccurate because they cluster types of behaviors that do not necessarily belong together. Future research should therefore consider whether more differentiated questions are needed.

## Conclusion

One key initiative to reach net-zero emissions are changes in energy production and consumption, which has been estimated to account for about 75% of the EU’s overall GHG emissions [82]. Our results indicate that high levels of personal and collective outcome expectancy strengthen the relationship between worry and energy curtailment behaviors, while high levels of collective outcome expectancy strengthen the relationship between worry and energy efficiency behaviors. Notably, believing that energy-saving can help reduce climate change does not seem to relate to curtailment or efficiency behaviors for those who are not at all worried about climate change. Based on these findings, campaigns aiming to lower household energy use could communicate the potential positive mitigation impact of individual and collective efforts to save energy, while simultaneously placing an emphasis on making people aware of the risks associated with climate change. If targeting groups that are not likely to worry about climate change, factors other than climate change mitigation, such as lowering the energy bill, might be more effective in motivating energy behaviors. However, conclusions should be made with caution as only a small portion of the variance in energy-saving behaviors was explained by the models.
